# Nuisance Ecology: Do Scavenging Condors Exact Foraging Costs on Pumas in Patagonia?

**DOI:** 10.1371/journal.pone.0053595

**Published:** 2013-01-03

**Authors:** L. Mark Elbroch, Heiko U. Wittmer

**Affiliations:** 1 Wildlife, Fish, and Conservation Biology, University of California Davis, Davis, California, United States of America; 2 School of Biological Sciences, Victoria University of Wellington, Wellington, New Zealand; University of Otago, New Zealand

## Abstract

Predation risk describes the energetic cost an animal suffers when making a trade off between maximizing energy intake and minimizing threats to its survival. We tested whether Andean condors (*Vultur gryphus*) influenced the foraging behaviors of a top predator in Patagonia, the puma (*Puma concolor*), in ways comparable to direct risks of predation for prey to address three questions: 1) Do condors exact a foraging cost on pumas?; 2) If so, do pumas exhibit behaviors indicative of these risks?; and 3) Do pumas display predictable behaviors associated with prey species foraging in risky environments? Using GPS location data, we located 433 kill sites of 9 pumas and quantified their kill rates. Based upon time pumas spent at a carcass, we quantified handling time. Pumas abandoned >10% of edible meat at 133 of 266 large carcasses after a single night, and did so most often in open grasslands where their carcasses were easily detected by condors. Our data suggested that condors exacted foraging costs on pumas by significantly decreasing puma handling times at carcasses, and that pumas increased their kill rates by 50% relative to those reported for North America to compensate for these losses. Finally, we determined that the relative risks of detection and associated harassment by condors, rather than prey densities, explained puma “giving up times” (GUTs) across structurally variable risk classes in the study area, and that, like many prey species, pumas disproportionately hunted in high-risk, high-resource reward areas.

## Introduction

Successful animals forage in ways that minimize intra- and interspecific competition and predation risk [1]–[4]. Interference competition describes the energetic costs associated with losing resources to another forager [1], [5], and predation risk describes the energetic cost an animal suffers when making a trade off between maximizing energy intake and minimizing the threat of an encounter with a predator [2], [6], [7]. Predation risk is an energetic cost not necessarily correlated with mortality [8], [9], but more broadly describes foraging costs associated with both direct predation and indirect effects associated with predator avoidance behavior [10].

Observed spatial variation in predation risk has been used to define *landscapes of fear* [4], [10], [11], and to test predictions made by foraging theories (e.g. [12]). Experimental manipulations of supplementary food sources and an animal's “giving up densities,” or GUDs, have been the predominant method to document and quantify habitat specific predation risk in natural systems [10], [13]. In predation risk experiments, researchers equate food abandoned by a forager (i.e., GUDs) as a metric of foraging risks in the environment. The less food remaining at a feeding station (e.g., lower GUD), the safer the animal felt while feeding [10]. To date, predation risk has rarely been quantified for carnivores. The few studies attempting to quantify predation risk for carnivores, focused on risks of medium sized carnivores being killed by larger, dominant species, rather than GUDs (e.g., cheetah, *Acinonyx jubatus* [14], *and* red foxes, *Vulpes vulpes* [15]). Whereas negative fitness consequences for carnivores have also been attributed to scavenging (e.g., [16], [17]), here, we evaluate whether carnivores suffering from scavengers exhibit behaviors comparable to those of prey foraging in a risky landscape.

Kleptoparasitism, the stealing of food from another animal, is a common form of interference competition [18]. Kleptoparasitism tends to increase in frequency when food resources are scarce, search time for food is high, or if there is asymmetry between the two competitors–meaning that larger, more powerful kleptoparasites more easily and more often steal from physically inferior species or conspecifics [18], [19]. Vertebrate scavengers are generally considered opportunistic foragers of available resources rather than true competitors (see review in [20]), and therefore researchers have assumed that unlike predators, scavengers do not exhibit top-down control on prey populations [21]. However, if scavengers did affect predator foraging patterns, scavengers could have significant indirect influences on community structure and composition.

The puma (*Puma concolor*) is a solitary felid and the top predator throughout Patagonia, a vast 1,000,000 km^2^, sparsely populated region below latitude 39°S in southern Chile and Argentina [22]. The Andean condor *(Vultur gryphus)* is an IUCN near-threatened species [23], and a large, diurnal, avian scavenger sympatric with pumas throughout the year over most of the pumas' range in southern South America. Condors are scavenging kleptoparasites, hunting only compromised prey (e.g., sheep, *Ovis aries*, hooked on fences) or newborn ungulates [24, Elbroch pers. obs.]. Condors are clumsy on the ground and forage only in open habitats [25]. Although condors are much smaller than pumas (8–15 kg, Houston [25] vs. 32–82 kg, [26]) and physically inferior, we observed high levels of condor scavenging from puma killed-ungulates and multiple condors at each carcass in Chilean Patagonia [27]. Thus, we hypothesized that kleptoparasitism by condors might influence puma foraging decisions, thereby altering puma kill rates and potentially influencing community structure and local biodiversity in Patagonia. We set out to answer three questions: 1) Do condors exact a foraging cost on pumas?; 2) If so, do pumas exhibit behaviors that betray their awareness of these risks?; and 3) Do pumas exhibit behaviors typically associated with species foraging in risky environments?.

To address question 1 and determine whether condors exact a foraging cost on pumas, we tested whether condor scavenging was associated with increased puma kill rates. In terms of optimal foraging theory [12], we hypothesized that scavenging by Andean condors would decrease handing times [28] thereby forcing pumas to increase kill rates to meet energetic needs. Further, because condor scavenging occurs throughout the year, we hypothesized that puma kill rates in Patagonia would be higher than those reported for North America [29], where avian scavengers eat at slower rates [30] and large, terrestrial scavengers like bears (*Ursus* spp.) only exhibit a seasonal influence on puma foraging (e.g., the effect of brown bears, *U. arctos*, on Eurasian lynx, *Lynx lynx*, in [17]).

To address question 2, we used GUD-like data. Traditional GUD experiments use feeding stations and resource subsidies so that every station offers the same amount of food in an identical matrix [10]. With equal proportions, the remains of food can then be measured and used as a correlate for foraging versus vigilance. Because providing supplementary food to pumas under natural conditions posed significant difficulties, we used actual puma kills. Thus, we faced potential problems created by different-sized prey–meaning that the amount of meat abandoned by pumas was not entirely due to risk of harassment by condors, but confounded by the size of the prey. For this reason, we created an inverse-GUD-like index from time spent at the carcass. We used handling time at the carcass as a measure of what we called giving-up-time (GUT), a more direct way to measure quitting harvest rates but a coarser metric with which to determine the tipping point between the influence of resource density and the costs associated with foraging in areas where puma kills are likely to be detected by condors. Foraging theory predicts foragers should have higher giving up densities in areas where resources are most abundant *and* where risk of detection by predators is highest [10]. Using GUT data, we tested these two hypotheses to determine whether patterns of meat abandoned by free-ranging pumas were better explained by 1) relative prey densities (resource abundance) or 2) risk of detection and harassment by condors, and the subsequent abandonment of carcasses.

To determine if pumas exhibit behaviors typically associated with species foraging in risky environments, we predicted that risk of detection by condors would explain variable GUTs for pumas on the landscape, and that pumas would disproportionately hunt in the riskiest landscapes with the highest prey densities, where resource rewards might compensate for losses to scavengers [3], [31].

## Materials and Methods

### Study site

**Figure 1:**
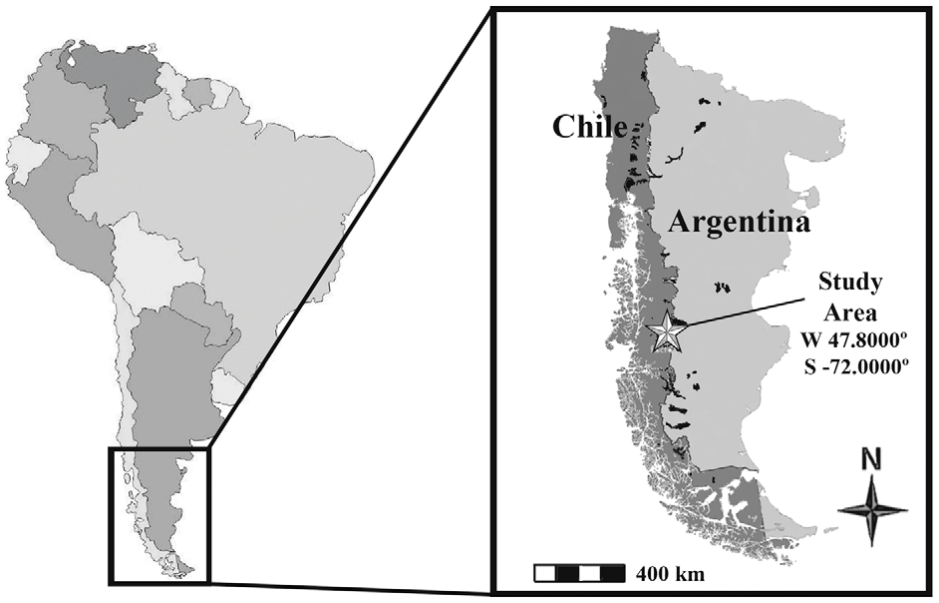
Location of the study area in Chilean Patagonia in southern South America.

Our study was conducted in the southern portion of Chile's Aysén District, immediately north of Lago Cochrane in central Chilean Patagonia (W 47.8000°, S −72.0000°, Fig. 1). The study covered an area of approximately 1063 km^2^ comprised of 5 distinct habitat types as defined in Elbroch and Wittmer [26]: valley steppe (74 km^2^), mountain steppe (754 km^2^), shrubs (138 km^2^), forests (44 km^2^), and barren mountaintops (53 km^2^). Valley steppe was flat steppe habitat 200–600 m asl distributed along the floor of Valle Chacabuco, and characterized by unobstructed views. Mountain steppe described steppe habitats 600–1,200 m asl, and was characterized by steep slopes, ravines, and rock outcrops providing pumas greater cover than valley steppe. Shrub habitats occurred between 200–700 m asl, and were characterized by dense woody understories and canopies up to 8 m tall. Forests habitats were found between 700–1,200 m asl, and were dominated by lenga (*Nothofagus pumilio*), had relatively open understories, and closed canopies >12 m tall. Barren mountaintops were found above 1,200 m asl, held limited vegetation <6 cm tall, but included rugged topography similar to that described for mountain steppe.

The study area supported large numbers of puma prey, including approximately 6,500 native guanacos (*Lama guanicoe*), 120 endangered huemul (*Hippocamelus bisulcus*), 22,000 European hares (*Lepus europaeus*), and 2,500 domestic sheep [26]. We characterized densities of guanaco, huemul, sheep, and European hare in the 5 principal habitats [26] to quantify total kg of prey available in each habitat type: 6,042–16,019 kg/km^2^ in the unfenced portions of valley steppes versus 5,081–5,357 kg/km^2^ in sheep enclosures in valley steppes; 3,635–9,645 kg/km^2^ in mountain steppes, 177–716 kg/km^2^ in shrub habitats, 121–477 kg/km^2^ in forests, and 0–50 kg/km^2^ on barren mountain tops. Culpeo foxes (*Lycalopex culpaeus*) and several scavenger birds, including Andean condors, southern (*Polyborus plancus*) and Chimango caracaras (*Milvago chimango*), and black-chested buzzard eagles (*Geranoaetus melanoleucus*) were common.

### Captures, collar programming, and ethics statement

Our capture procedures adhered to guidelines suggested by the American Society of Mammalogists [32], were approved by the independent Institutional Animal Care and Use Committee at the University of California, Davis (Protocol # 13252), and were conducted under permit # 6267 issued by the Gobierno de Chile, Ministerio de Agricultura (SAG, Chile Agricultural and Cattle Service). We captured pumas from March to September in 2008 and 2009, when locating them was facilitated by the presence of snow on the ground. When conditions were suitable, we traveled on horseback until fresh puma tracks were found, and used hounds to force pumas to retreat to either a tree or rocky outcrop where we could safely approach an animal. Pumas were anesthetized with Ketamine (2.5–3.0 mg/kg) administered with a dart gun, and then lowered to the ground where they were administered Zalopine (0.075 mg/kg) by syringe. We fitted pumas with either an Argos-GPS collar (SirTrack, Televilt, or Lotek) or VHF collar (SirTrack), the weights of which were less than 3% of adult female weights in the study area, and less than 2% of the weight of adult males. Once an animal was completely processed, the effects of the capture drugs were reversed with Atipamezole (0.375 mg/kg), and pumas departed the capture sites on their own.

GPS collars were programmed to acquire location data at 2-hr intervals and transmit data through an Argos uplink at 2–5 day intervals. Our most common programming was a 6-hr Argos uplink every 3 days. The numbers of kittens for collared females were determined during captures of their mothers, direct observations, by tracks in snow, and/or remote cameras at active kill sites.

### Field investigations

Upon retrieval, location data were displayed and distances between consecutive puma locations were calculated in ArcGIS 9.1. (ESRI, Redlands, CA). We defined GPS clusters [33] as any ≥2 locations within 150 m of each other, and CyberTracker-certified observers [34], [35] conducted field investigations of any cluster where at minimum 1 GPS location was recorded during crepuscular or nocturnal periods, the exact timing of which varied with season. For the first 2 months of the project, we investigated every cluster, even those made completely within daylight hours. Because daytime clusters did not reveal a single predation event and required significant time to investigate (each puma would make 1–3 short daytime clusters per day), we chose not to investigate clusters with durations completely within daylight hours for the remainder of the project and to assume that they represented resting rather than kill sites. Observers located areas associated with clusters by transferring location data to handheld GPS units (Garmin eTrex and Summit models) used to guide them in the field.

Prey remains, including hair, skin, rumen, and bone fragments, were used to identify prey species, and the state of prey remains, including the location of bite marks and body parts consumed, were used to determine whether the puma had killed the animal or was scavenging. For ungulates, we defined the kill site (recorded with GPS, accuracy 5–10 m) as the location where the rumen was found. The age of guanacos up to 24 months old were determined using tooth eruption sequences in the lower mandible [36]. The monthly weights of 1-year (chulengos) and 2-year guanacos were estimated using linear growth estimates, a birth weight of 12.7 kg, and 1-yr and 2-yr weights of 42 kg and100 kg, respectively [37]. Guanacos >2 years of age were considered adults and to weigh 120 kg [36]. Data were not available for growth rates in huemul, excepting estimated birth weights of 5 kg [38]. Instead, we applied growth allometry for the structurally similar mule deer (*Odocoileus hemionus*), which gain weight at a rate of 0.21 kg/day [39]. Annual huemul weights up to 3 years were estimated based on growth rates reported for mule and white-tailed deer (*O. virginianus*) [40]. We used adult huemul (>3 years) weights of 65 kg [41]. The ages of huemul up to 3.5 yrs old were estimated using tooth eruption sequences. For small prey, we assumed 4 kg for European hares (the mean weight of 30 specimens hunted by locals or killed by vehicles in our study area), 2 kg for Patagonian haired armadillos (*Chaetophractus villosus*) [41], 9 kg for culpeo foxes [41], and 6.4 kg for Upland geese (*Chloephaga picta*) [42].

We documented whether each carcass was cached (covered with debris), but did not differentiate between caching efforts (e.g., whether the carcass was partially or completely covered). Canopy cover at puma kills was measured with a convex spherical crown densiometer (Forestry Supplier, Kackson, MS). Each densiometer delineated 24 squares with which to quantify canopy cover, and we subdivided each square into 4 quarters, allowing for a potential of 96 total units. With the densiometer held directly over the kill, the canopy was quantified as % canopy cover, calculated as the variable number of 96 sections in which vegetation was visible.

We recorded the presence of condors at puma kills through direct sightings, using remote cameras at kills, and/or through associated signs, including feathers, droppings and footprints. We did not attempt to determine whether condors were present at kills investigated more than two weeks after the puma left the area, when signs were more difficult to interpret.

### Defining handling time and search time

We defined the handling time as the total hours from the first to the last GPS location recorded by the puma collar within 150 m of the kill site, even when the puma moved away from the kill site and then returned during the duration of actively feeding at the site (e.g., traveled to a resting site in a different location or to retrieve kittens and escort them to the kill). We only quantified handling time for the subset of kills in which Argos and GPS performance allowed us to accurately identify the start and end of each kill, which required continuous GPS locations leading up to and away from a kill.

We defined the search time as the total hours between the last GPS location within 150 m of a kill site and the first GPS location within 150 m of the subsequent kill site. Thus we divided puma activity into 2 phases: searching and handling, choosing not to differentiate from among the other behaviors that occurred within each phase (e.g., resting). For kill sequences in which Argos and GPS performance allowed us to accurately identify the last point of the first kill and the first point at the next kill, we calculated the distance pumas moved between sequential kill sites in two ways. First, we summed the straight-line distances between sequential locations gathered by the GPS collars for the duration of the search time. Second, we corrected the summed straight-line distances by applying a correction factor of 1.64, calculated by walking a subset of random 2-hr segments of puma trails to better estimate the distances pumas travel in the field [26]. We tested assumptions of ANOVA [43] for our search time and distance data. A square-root transformation was applied to the search time data and a logarithimic transformation to the search distances, after which both data sets exhibited normal distributions. We used mixed model ANOVAs (SAS 9.2, Cary, NC) to incorporate puma as a random effect and to account for variable samples across individual pumas. We tested whether sex, and/or the presence of kittens among females explained variation in search time and/or inter-kill distances.

### Calculating kill rates

We calculated kill rates using two different methods. First, we quantified total kill rates (kg/day) for all prey killed for pumas monitored continuously for a minimum of four weeks. Second, we calculated ungulate kill rates (excluding all small prey) for the same time periods, for comparison with kill rates for pumas in North America published in Knopff et al. [29]. Using data collected during the same time periods, we also determined the amount of time (hrs) that pumas were located near known kill sites (handling time) and the amount of time they were in between known kill sites (search time) to calculate a percent time associated with feeding sites (time associated with kills/total time of monitoring). We did not include any periods in which Argos transmissions missed GPS locations for ≥1 night. For pumas in which there was a gap in monitoring, and thus two periods of continuous monitoring greater than four weeks in length, we calculated kill rates for each period separately. We tested for differences in kill rates among males and females and among females with and without kittens using mixed-model ANOVAs that incorporated individual pumas as a random effect to account for variable samples across pumas.

### Testing whether scavenging by condors influenced puma kill rates

We used a multivariate approach to test whether prey weight (in kg), the presence of condors, canopy cover, and whether the kill was cached carried predictive power in determining handling time. We used pairwise coefficient correlations, and a correlation cut off of 0.5, to test for significant correlation between independent variables. The presence of condors and canopy cover proved significantly correlated, and we selected the presence of condors to be included in the final analysis because it yielded the highest correlation with handling time. In the end, three variables remained to run the model, and we included individual pumas as a random effect to account for variable samples across pumas. Because the data were discrete, we employed a generalized linear model (GLIM) and a Poisson distribution (logarithmic link function) to account for the fact that time at a carcass could not be negative.

We then used a GLIM to test whether handling time or prey weight explained variation in the search time immediately following that kill (i.e., did a puma that spent less time at a carcass exhibit a shorter search time before its next kill in sequence than did a puma that spent more time at a carcass). We incorporated puma as a random effect to account for variation in prey availability among pumas and for variable number of samples from different animals. We repeated the analysis twice, first with all prey data, and second with only the ungulate prey data to re-assess the relationship in case small prey might have biased results.

### Quantifying risk of harassment by condors and prey abundance, and testing their effect on GUTs

For every carcass that a puma abandoned >10% of the edible meat, we defined the GUT as the handling time (determined from location data gathered at 2-hr intervals). We used a >10% threshold because it increased the sensitivity of our method by allowing us to include smaller ungulate prey that could be eaten more quickly. We expected that any effects on GUTs that we detected at kills with >10% meat remaining would be magnified should we limit our analysis to kills at which pumas abandoned a larger amount of meat. Based on our definition, the smallest GUT was 2, where a puma remained for 2 hours, and the largest GUT was 232, which was the longest period a puma was associated with a carcass. While our measurement may at first seem counterintuitive because traditionally, the larger the GUD, the greater the risk, in our research, the smaller the GUT (the shorter the time at the carcass), the greater the implied risk.

**Figure 2:**
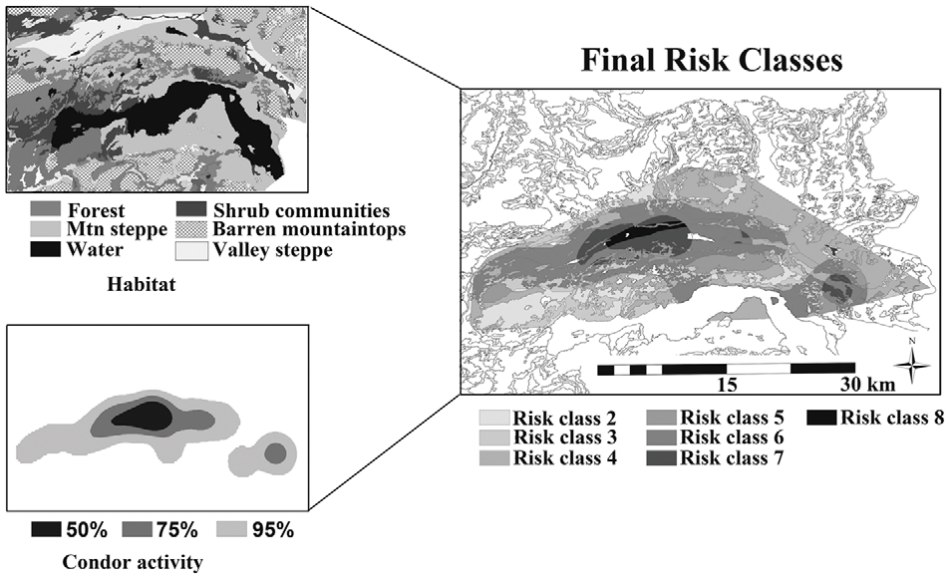
Habitat classes, fixed kernel condor activity polygons, and final risk classes in our 1,062 km^2^ study area created through the union of the two layers.

We defined risk classes categorically, by combining two risk scores. The first score reflected the relative risk of harassment by condors we assigned the 5 habitat types found in the study area, and the second score reflected apparent risk as quantified by the spatial distribution of condor activity on the landscape. We first calculated a correlation coefficient of these two variables and determined they were independent (coefficient  = 0.04). Habitats were assigned risk factors (lowest to highest, 1–4) based upon carcass visibility for condors (habitat descriptions are found in the study area description). We assigned forests a risk of 1, shrubs 2, open mountain steppe and Barren mountaintops 3, and open valley steppe 4. We then plotted the locations of kills detected by condors and used a fixed kernel density estimator [44] to create 50%, 75% and 95% condor activity polygons (*sensu* predation risk in [45]). The kernels were created using least square cross validation (LSCV) to determine the smoothing factor *h* [46], and the Animal Movement Extension [47] in ArcView 3.2 (ESRI, Redlands, CA). We assigned a risk of 1 for kills that fell outside the 95% kernel for condor activity, 2 for kills that fell within the 95% kernel, 3 for those within the 75% kernel and 4 for kills inside the 50% kernel to account for increasing risks of harassment by condors. We then combined the two layers (habitats and condor activity) using the union function in ArcGIS 9.3 to create polygons reflecting both habitat type and condor activity (Fig. 2). We defined the risk class for a given area as the as the mathematical sum of the risk associated with habitat and the risk associated with condor activity (range of 2 to 8).

We calculated prey densities for each habitat type using direct counts for huemul and sheep, and Distance sampling [48] for guanacos and hares (details presented in [26]). We defined both risk and prey density (low for forests and barren mountaintops, medium for shrubs, high for mountain steppes, and highest for valley steppes) as categorical data (see study area description for discussion of prey densities in each habitat). We used a mixed-model ANOVA with puma as a random effect to test whether prey density or habitat specific risk categories had greater explanatory power for GUTs. We then used an ANOVA and Tukey-Kramer test to determine whether mean GUTs varied across different risk classes. Finally, we used a chi-square goodness of fit test to assess whether large animals killed by pumas in which >10% of edible meat was abandoned, were distributed across risk classes in proportion to their availability in the study area.

## Results

### Prey indices and GPS cluster characteristics

We monitored 8 pumas using Argos-GPS technology (1 SirTrack, 7 Lotek Wireless), and 1 puma using the stored GPS data in the collar because its Argos capabilities failed (Telonics collar), for a mean of 9.33±5.66 months. We conducted field investigations of Argos-relayed GPS clusters within 11±12 (range, 0 – 78) days of the time the puma left the area. We did not use the stored data in the malfunctioning collar in this calculation; site investigations for this animal were conducted on average 792±87 (range, 650 – 945) days after the puma had left the area [49]. We investigated 694 GPS clusters, and identified 433 Kill Sites and 6 acts of puma scavenging. Prey included 350 ungulates (7 huemul deer, 41 domestic sheep, and 302 guanacos), and 83 small to medium-sized vertebrates. More information on prey indices can be found in Elbroch and Wittmer [49].

Pumas abandoned an estimated 29±27 kg of meat at each ungulate kill (range 0 – 75 kg). We documented condors at 43% of 296 ungulate kills in which their presence could be determined with certainty. For kills in which handling time could be quantified, pumas abandoned 133 of 266 ungulate kills (or 50%) where >10% of edible meat remained after 1 night, and an additional 56 (or 21%) after 2 nights.

### Search times and distances

Mean search time for all prey was 70.2±51.4 hrs (*n* = 340), and 69.5±51.4 hrs for only ungulate kills (*n* = 229). Neither sex (*F*_1,4.74_  = 0.85, *P* = 0.40) nor the presence or absence of kittens with females (*F*_1, 4.09_  = 1.32, *P* = 0.31) were significant predictors of search time. The mean distance traveled between sequential kill sites was 19.83±19.23 km (32.52±31.55 km when a correction factor was applied). Males and females moved similar distances (*F*_1,3.88_  = 03.44, *P* = 0.14), as did females with and without kittens (*F*_1, 3.5_  = 1.79, *P* = 0.26).

### Kill rates

One male puma (M1) moved into Argentina before 4 weeks of continuous monitoring [50] and was excluded from the kill rates analyses. Poor Argos performance limited our ability to monitor remaining pumas continuously for the entire duration they were collared. Monitoring periods are listed in Table 1. Kill rates did not differ between sexes (*F*_1,6_ = 0.08, *P* = 0.78 Total kill rates for all prey were 12.64±5.71 kg/day, and ungulate kill rates were 12.49±5.81 kg/day. The mean time pumas were associated with kill sites, defined as the time during which they were actively feeding or traveling between visits to a kill site, was 28.7±7.5% (Table 1). The remainder of their time was spent between consecutive kill sites, which we defined as search time.

**Table 1:** Individual puma kill rates and the % time pumas were associated with active kill sites.

	Length of continuous monitoring (days)	Total No. of Kills	Total Kg of prey killed	Kg prey killed per day	No. ungulates killed	Total Kg of ungulates killed	Kg ungulates killed per day	% of time associated with Kill Sites
M2	45	7	785	17.4	7	785	17.4	32%
M2	30	5	600	20	5	600	20	28.30%
M3	164	40	2114	12.9	40	2114	12.9	17.00%
M3	120	34	1754	14.6	34	1754	14.6	16.50%
M4	79	10	622	7.9	10	622	7.9	24.70%
F1	202	38	2991	14.8	38	2991	14.8	34.10%
F2	62	10	621	10.0	9	617	10.0	23.70%
F3	169	50	3617	21.4	42	3585	21.2	37.50%
F4	421	110	4415	10.5	74	4279	10.2	37.90%
F5	208	53	837	4.0	22	713	3.4	25.00%

### Influence of condors on handling or search time

Prey weight (*F*_1,265_  = 18.11, *P*<0.0001), the presence of condors (*F*_1,265_  = 8.17, *P* = 0.0046), and whether the kill was cached (*F*_1,265_  = 6.35, *P* = 0.0123) all proved significant influences on handling time. The model explained 63% of the variation in handling time, of which prey weight explained 18%, the presence of condors, 14%, and whether the kill was cached, 12%. Handling time increased with prey weight and caching behaviors, but decreased with the presence of condors.

Shorter handling times correlated with shorter search times in both the analysis using all prey (*F*_1,335_  = 3.67, *P* = 0.05) and the analysis using only ungulate prey (*F*_1,245_  = 2.13, *P* = 0.04). Prey weight did not affect search time for the ungulate data (*F*_1,255_  = 0.91, *P* = 0.43) or prey species combined (*F*_1,316_  = 0.26, *P* = 0.50).

### Results of GUT analysis and kill locations in a risky landscape

GUTs differed among risk classes (*F*_6,205_ = 5.60, *P*<0.0001), and were significantly smaller in the areas of highest risk of harassment by condors (Table 2). Using 212 kills where the puma abandoned >10% of the edible meat, total risk was significantly associated with GUTs (*F*_6,199_  = 2.22, *P* = 0.04) whereas relative prey density was not (*F*_4,200_ = 0.61, *P* = 0.66). Puma kills were distributed disproportionately among the riskiest areas more than expected given their distribution in the study area (_6  = _182.4168, *P*<0.0001) (Table 3). Kills were located 4–5 times more often than expected in polygons ranked in the two riskiest categories.

**Table 2:** Mean GUTs for each risk class 2–8, and results of Tukey-Kramer test.

Risk Class	No. of kills	GUT ( = Handling time)	+/− SD	Risk classes that share the same Letter are statistically equivalent.			
2	4	93.5	21.46	A	B		
3	11	51.1	12.941	A	B	C	
4	29	63.4	7.970	A			
5	52	48.5	5.952	A	B	C	
6	33	26.1	7.471		B	C	D
7	60	24.0	5.541				D
8	23	20.9	8.949			C	D

**Table 3:** Observed versus expected distributions of large kills across Risk classes.

Risk category	Habitat-Condor activity combinations in this risk class	Kills located in polygons	Observed frequency (%)	Expected frequency (%)
2	a) Forest-Low	7	0.3	13.3
3	a) Shrub-Low b) Forest-Medium	14	6.6	12.3
4	a) Shrub-Medium b) Forest-High c) Mountain steppe-Low d) Barren-Low	25	11.8	32.4
5	a) Shrub-High b) Forest-Extreme high c) Mountain steppe-Medium d) Barren-Medium e) Valley steppe-Low	63	29.7	23.9
6	a) Shrub-Extreme high b) Mountain steppe-High c) Barren-High d) Valley steppe-Medium	29	13.7	10.6
7	a) Mountain steppe- Extreme high b) Barren-Extreme high c) Valley steppe-High	58	27.4	5.8
8	a) Valley steppe-Extreme high	16	7.5	1.7

## Discussion

Our research provides evidence that scavengers exact foraging costs upon top predators, and that top predators are aware of and responsive to the costs associated with detection and harassment by competitive scavengers. The fact that pumas abandoned >10% of the edible meat at 50% of large carcasses they killed after a single night of feeding, and never returned to the carcass to see whether meat remained to be eaten, was strong evidence that pumas were aware of the inherent risks of losing carcasses to condors in open habitats. The additional abandonment of 21% of large carcasses with >10% edible meat remaining after only two visits further supported this argument.

Puma kill rates for ungulates only (in kg/day) in our study area (12.49 kg/day) proved 50% greater than those reported by Knopff et al. [29] for pumas in North America (8.28 kg/day). Condor scavenging significantly decreased the handling time of pumas in our study area, presumably because pumas wanted to avoid condor harassment. Shorter handling times were associated with shorter search times before pumas killed again (e.g., higher kill rates), but search times were not influenced by prey weight at the most recent kill. Consequently, our analyses suggested that kill rates in our study area were increased because pumas abandoned their kills more frequently to avoid harassment by scavenging condors.

Pumas in North America minimize losses to bears and gray wolves (*Canis lupus*) by shifting their activities into more structured environments and avoiding open habitats where they are most at risk of losing a kill [51], [52]. The longest GUTs in our study pertained to kills made in forests (risk categories 2–4 in Table 2), indicating that forests in Patagonia also offered the greatest protection against harassment by condors and other competitive scavengers. Unlike in North America, however, where ample puma prey exists in forests [53], prey biomass in Patagonia is located almost entirely in open grassland habitats [26]. Caching behaviors by pumas in our study mitigated potential losses to scavengers, but delayed rather than stopped detection of kills by condors in open habitats.

Individual condors can ingest approximately 1.5 kg of meat per feeding bout (*sensu* whiteback griffon vulture, *Gyps africanus,* [54]), and in our study area, condors fed in groups of up to 28 individuals (Elbroch pers. obs.). Thus, once a large carcass was detected, groups of foraging condors were capable of consuming entire carcasses in just several hours. We only once observed Andean condors challenging a puma for a carcass. Instead, condors took advantage of food left undefended in open areas. The puma we did witness actively defending its kill from condors was unsuccessful in defending simultaneous attacks from multiple fronts, and eventually abandoned the carcass to the condors.

The question remains, however, as to why pumas abandoned their kills in the first place. We have discussed the avoidance of condor harassment as the potential mechanism driving this behavior–perhaps mobbing condors are difficult to defend against for a solitary predator. Alternatively, condors might not be driving the behavior, but taking advantage of abandoned carcasses. Historic puma persecution in Patagonia was so high as to result in puma extirpation through most steppe grasslands [22], [55]. Open habitats not only make puma kills more vulnerable to detection by condors, but also the pumas themselves to detection by hunters and ranchers. Yet, regardless of whether fear of humans or the threat of harassment by condors drives pumas to leave carcasses in open areas before they are consumed, our data suggest that pumas know not to return to their kills in open habitats because condors will have consumed the remainder of the edible meat. Thus, condors ultimately exact the foraging cost.

It is particularly important that puma GUTs were better explained by relative risk of harassment by condors than by relative prey density. This is strong evidence that top predators suffering energetic costs associated with kleptoparasites exhibit behaviors similar to prey species foraging in *landscapes of fear* [11]. As we predicted, pumas also killed disproportionately more ungulate prey and abandoned the most food in the riskiest areas with the highest prey densities–this incongruity between foraging costs and resource availability has been observed with many species that forage in risky environments with variable resource availabilities (e.g., [3], [31]). The benefits of foraging in disproportionately resource-rich areas presumably trump any costs associated with foraging there, and thus many species select high-risk, high reward areas in which to forage [3]. In the case of pumas in Patagonia, where the risks associated with scavenging condors were but a nuisance, the costs of foraging in a high-risk area for pumas may have been more easily absorbed when compared with species in which predation risk frequently equates to death. When pumas abandoned their kills in open habitats, however, they suffered an energetic cost both in losing calories to kleptoparasites and in expending additional calories to hunt more frequently. This raises important questions for future research as to whether humans or condors are driving the initial abandonment of kills, and at what level nuisance condors affect puma survivorship or recruitment?.

We believe our work also lends important insights for understanding predator-prey theory and population dynamics of endangered prey species affected by predation. Our results support previous suggestions that simplistic single prey-single predator models are unable to accurately predict the dynamic consequences of predator-prey interactions in natural systems (e.g., [56], [57]). In our study area, scavengers may also be indirectly affecting the composition of communities and ecosystems by increasing the top-down control of pumas on their prey. We have shown that pumas in our system that abandoned kills to condors exhibited higher kill rates than pumas in North America, and exhibited shorter handling and search times. As a consequence, pumas with higher kill rates also exhibit higher encounter probabilities with their prey. An overall increase in search time and increased prey encounters over longer time periods could be particularly problematic for rare prey species, and in our system may at least partially explain current unsustainable mortality rates of endangered huemul from puma predation [49], [58].
